# A Solar Water‐Heating Smart Window by Integration of the Water Flow System and the Electrochromic Window Based on Reversible Metal Electrodeposition

**DOI:** 10.1002/advs.202104121

**Published:** 2021-12-28

**Authors:** Ling Wang, Xiuling Jiao, Dairong Chen, Ting Wang

**Affiliations:** ^1^ National Engineering Research Center for Colloidal Materials School of Chemistry and Chemical Engineering Shandong University Jinan 250100 China

**Keywords:** electrochromic, energy storage, reversible metal electrodeposition, smart windows, water flow windows

## Abstract

Various smart windows with dynamic modulation of the light transmittance have been developed rapidly in recent years. However, current design of the smart windows can only modulate the indoor solar irradiation instead of effectively utilize them. Here, a solar water‐heating (SWH) smart window is proposed by the integration of the traditional electrochromic window and the water flow system, which can not only provide dynamic daylight modulation but also harvest the solar energy and store them by heating water. In the SWH window, the reversible metal electrodeposition (RME) not only provides daylight modulation but also provides metal layer working as a flat‐plate solar collector for energy harvesting. Compared with traditional electrochromic windows, the SWH window with a water flow system can more effectively modulate the indoor temperature, owing to the significantly enhanced tunability of the thermal irradiation from the window. Compared with water‐flow windows, the RME provide a metallic layer for efficient light harvesting, up to 42% solar energy can be effectively harvested and stored as hot water. Such an SWH smart window is promising to reduce the heating, lighting, and air conditioning energy consumption, which may bring new insights in the design of the next‐generation green buildings.

## Graphical abstract

A new design of solar water heating smart window with the integration of the traditional electrochromic window and a water‐flow system is proposed. Light transmittance, room temperature control, and energy collection/storage can all be simultaneously modulated in such a window, which provides energetic and functional advantages over the traditional electrochromic windows and the water‐flow windows.

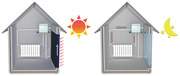

## 1 Introduction

**Figure 1:**
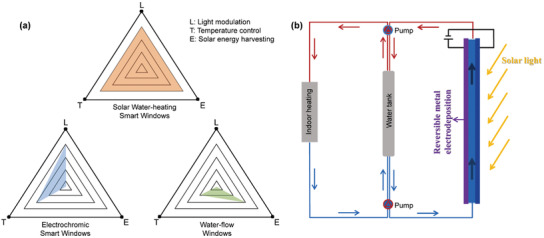
a) Performance parameters for three different designs: SWH smart windows (orange), electrochromic windows (blue), and water‐flow windows (green). b) Schematic illustration of the new solar water‐heating smart windows.

The usage of building energy, such as heating, ventilation, and air conditioning, accounts for 40% of the total energy consumption, and heating accounts for ≈75% of the energy demand within the buildings.^[^ ^1^ ^]^ Smart windows, which can dynamically modulate light transmittance, are recognized as a promising technology to economize building energy consumption.^[^ ^2^, ^3^ ^]^ Among various types of smart windows, electrochromic windows provide dynamic control of both the light and heat flow into and out of buildings, meanwhile, the view through the glass can be maintained, thus providing energetic and functional advantages over static controls (such as blinds or shades). Compared with traditional electrochromic materials, such as transition metal oxides, polymers, and small organic molecules, the reversible metal electrodeposition (RME) strategy is highly promising and rapidly developed in recent years. With Bi‐Cu bimetal electrodeposition and nickel oxide as the counter electrode, the RME window can be uniformly switched between a transparent state and a color‐neutral black state within a minute.^[^ ^4^ ^]^ With proper selection of the electrolytes, such as the acidic perchlorate electrolytes, long‐term durability up to 10 000 stable cycles can be obtained.^[^ ^5^ ^]^ With proper polymer inhibitors, RME window with size up to 900 cm^2^ can be constructed.^[^ ^6^ ^]^ Owing to the rapid switching speed, long‐term durability, neutral color, and homogeneity, the reversible metal electrodeposition is promising to be used in practical large‐size smart windows.^[^ ^7^ ^]^ One interesting fact is that, for the RME process, with the metal deposited on the conductive substrate, as‐formed metal layer actually works as a flat‐plate solar collector, which efficiently converts solar energy into heat. However, the solar heat on the metal layers is uncollected and radiated both indoor and outdoors, which is a waste of valuable solar energy. Meanwhile, the solar heat radiated through the inner glazing of the window reduces the effectiveness for indoor temperature tuning.^[^ ^8^ ^]^ Currently, most of the electrochromic window design cannot utilize the thermal energy on the window panes, which deserves more investigation (**Figure** **1**a).

Other than the dynamic smart windows with tunable transparency, concepts such as water‐flow window water‐filled glass and fluidic windows have also been proposed.^[^ ^9^, ^10^, ^11^, ^12^, ^13^ ^]^ In a typical design, the cavity of a double pane window is connected to a water‐flow circuit, the solar heat at the window glasses can be readily collected and transported by the water stream and be stored in a water tank. The stored hot water can be used for indoor utilizations. In such a design, the water‐flow window actually functions as a solar water‐heating (SWH) device. However, such windows cannot modulate the light transmittance, also, owing to the high transparency of the window panes, the conversion efficiency of the solar energy is commonly low (between ≈5% and 15%), which prohibits their future applications (Figure 1a).^[^ ^14^, ^15^ ^]^

Here, we propose a new design to combine the electrochromic dynamic window and the water‐flow window, namely an SWH smart window. The metal layers deposited on the conductive substrates work as flat‐plate solar collectors, which efficiently convert solar energy into heat, and the water flow systems transport/store the thermal energy for indoor heating (Figure 1b). Such a design can achieve efficient light modulation, indoor temperature control and the solar energy harvesting simultaneously (Figure 1a). In a typical design, we deposit Zn‐Cu bimetals on bare indium tin oxide (ITO) as the metal layer, the prussian blue (PB) film on ITO substrate as the counter layer and the mixed ZnSO_4_/CuCl_2_ solution as the electrolyte. A SWH smart window with size up to 10 × 10 cm^2^ is constructed with uniform, fast color switching (60% optical modulation within 4 min). For the light control, the smart window can show four different states (a transparent state, a blue state, a black state, and a mirror state) upon the RME process on bare ITO and the metal‐ion insertion/extraction in the PB film. With the water flow system, the SWH window can more effectively modulate the indoor temperature, owing to the significantly enhanced tunability of the thermal irradiation from the window panes. Meanwhile, up to 42% of the solar energy can be converted/stored as thermal energy for indoor heating, indicating efficient solar energy harvesting. The SWH smart window based on RME provides energetic and functional advantages over the traditional electrochromic windows and the water‐flow windows, which may bring new insights in green building construction.

## 2 Results and Discussion

The construction of the solar water‐heating smart window relies on RME on the conductive substrates. Single type of metal ions usually suffers from inhomogeneous deposition and poor durability. Bimetallic electrolytic systems with Cu ions, such as Pb‐Cu, Bi‐Cu, or Sn‐Cu, have been proposed to design metallic dynamic windows.^[^ ^16^, ^17^ ^]^ Here, the Zn metal with high thermal conductivity(112 W (m k)^–1^) and good compatibility with Cu (convenient formation of Zn‐Cu alloys) is investigated for the Zn‐Cu layer in the RME process.^[^ ^18^ ^]^

**Figure 2:**
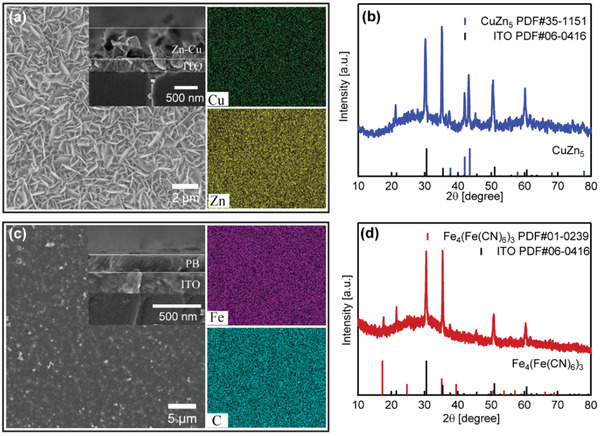
a) The SEM images and the corresponding EDS elemental mappings of the deposited CuZn_5_. b) The XRD pattern of the deposited CuZn_5_. c) The SEM images and the corresponding EDS elemental mappings of the deposited PB. d) The XRD pattern of the deposited PB film.

The Zn‐Cu metals were electrodeposited on conductive ITO substrates with an aqueous mixed solution of ZnSO_4_ (1 m)/CuCl_2_ ( 0.01m) as the electrolyte using an applied voltage of −1.0 V (vs Ag/AgCl) for 40 s. The scanning electron microscope (SEM) image (**Figure** **2**a) shows an array of thin nanosheets grown on the surface of the ITO substrates. The diameters of the nanosheets are ≈0.5–2 µm, the thickness of the array is ≈700 nm (inset in Figure 2a). The energy disperse spectroscopy (EDS) elemental mapping analysis demonstrates the nanosheet array contains homogeneously distributed Cu and Zn elements (the right part of Figure 2a). The X‐ray diffraction (XRD) pattern indicates the deposited nanosheets exhibit a phase of CuZn_5_ (powder diffraction file (PDF)#35‐1151) (Figure 2b). To further investigate the electrodeposition process, the cyclic voltammetry (CV) curves were obtained in a typical three electrode configuration with the Ag/AgCl as the reference electrode, the ITO substrate as the working electrode and Pt foil as the counter electrode. As shown in Figure S1a in the Supporting Information, the Zn‐Cu deposition shows features of Zn and Cu deposition individually.^[^ ^19^ ^]^ For the Zn‐Cu deposition, during the negative‐going sweep, a cathodic current appears at 0.01 V, which is attributed to the electrochemical reduction of Cu^2+^ to Cu^+^. The Cu^+^ ions combine with the chloride ions and forms complexes which are further reduced to Cu.^[^ ^5^, ^20^ ^]^ The cathodic current increases rapidly at −0.9 V owing to the reduction of Zn^2+^ to Zn. The anodic stripping peak centered at −0.68 V is caused by the dissolution of Zn, and the peak at 0.05 V corresponds to the dissolution of Cu.

The PB is a good electrochromic material with low operation voltage, high cycle stability, and rapid color switching, which is widely investigated for construction of smart windows.^[^ ^20^, ^21^ ^]^ Here, PB film was electrodeposited on bare ITO glass as the counter electrode with details provided in the Supporting Information. With the aqueous solution of ZnSO_4_ (1 m)/CuCl_2_ (0.01 m) as the electrolyte, the CV curve of the PB film shows a pair of peaks located at 0.7 and 0.95 V, corresponding to the reduction/oxidation of the hexacyanoferrates groups (Figure S1b, Supporting Information). The SEM image shows the PB film is homogeneously deposited on the ITO substrate with a thickness of ≈200 nm (Figure 2c, inset). The EDS elemental mapping analysis demonstrates homogeneous deposition of the PB layers (the right part of Figure 2c). The XRD pattern confirms the crystal structure of the deposited PB layers (Fe_4_(Fe(CN_6_)_3_, PDF#01‐0239, Figure 2d).

**Figure 3:**
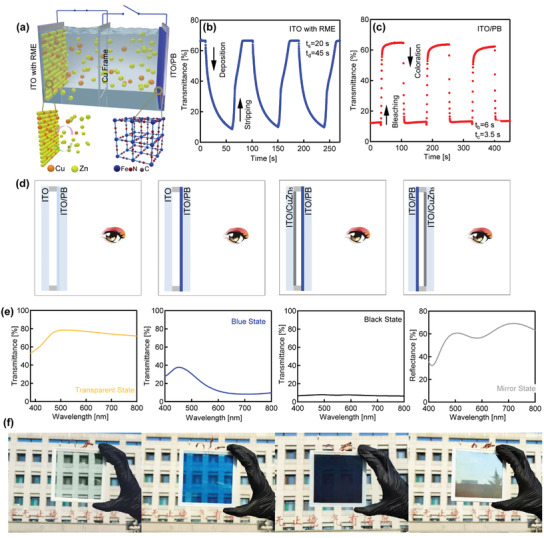
a) Schematic illustration of the device architecture. b) Transmittance measured at 700 nm versus time curves for the RME process with transmittance switching at voltages of 1.0 V (stripping) and −1.2 V (deposition). c) Transmittance measured at 700 nm versus time curves for the PB film with transmittance switching at voltages of 1.2 V (blue‐transparent) and 0.4 V (transparent‐blue). d) Schematic illustrations of the color states of the electrodes. e) Transmittance/reflectance spectra of as‐assembled device in different states. (f) Color images of as‐assembled device in different states.

A typical device is assembled by using two pieces of ITO glass: one bare ITO and one with deposited PB, and an aqueous electrolyte containing ZnSO_4_(1 m)/CuCl_2_(0.01 m) is filled between the ITO substrates. A copper frame is used as the third electrode for rapid color switching and device state modulation and the edges of the device are further sealed by 3D printed plastic frames (light‐sensitive resin) to avoid the electrolyte leakage (**Figure** **3**a and Figure S2, Supporting Information). For as‐assembled device with a size of 5 cm × 5 cm, dynamic optical transmittance measurements were performed at a wavelength of 700 nm and the transmittance dropped to minimum within 45 s by an applied voltage of 1.2 V between the bare ITO and the Cu‐frame. By reversing the current direction, the deposited metal layers dissolved in 20 s. An optical contrast up to 60% can be observed for the metal deposition/dissolving cycles (Figure 3b). For the counter PB films, by connecting the PB film with the Cu‐frame and applying a voltage of 1.2 V/0.4 V, the switching time between the blue state and the transparent state are 3.5 s (transparent to blue) and 6 s (blue to transparent) and the optical contrast between the two states is 54% (Figure 3c). The cycle stabilities of as‐assembled devices were obtained by monitoring the optical contrast between different states of the electrodes. As showing in Figure S3 in the Supporting Information, after 1000 cycles, the bare ITO with/without metal deposition still delivered a transmittance contrast up to 55% at 700 nm, and for the PB film, the transmittance contrast up to 40% can still be retained. These values indicate high stability of as‐assembled device after 1000 cycles. As shown in Figure S3b in the Supporting Information, for the cycle stability of the PB film, the performance dropped rapidly within 200 cycles, then gradually increased till 1000 cycles. From the obtained cyclic voltammogram results (Figure S4, Supporting Information), the ion diffusion coefficients (D) were calculated, and the greatly reduced *D* value within 200 cycles indicates possible lattice distortion, which may reduce the performance of the PB film. From 200 cycles to 1000 cycles, the *D* value only slightly changed, and the SEM shows the surfaces of the PB film were etched to nanoporous morphology (Figure S5, Supporting Information), which may increase the active surface area and promote the performance of the PB film.

With the same method, a device with a size of 10 cm × 10 cm was also assembled, and homogeneous deposition of metal layers can still be obtained, even though the color switching for the edge parts of the film is more rapid than the center (Figure S6 and Tables S1 and S2 in the Supporting Information). Up to now, the RME strategy can be used to assemble smart windows with size up to 900 cm^2^,^[^ ^2^, ^6^ ^]^ indicating a promising future in scale‐up fabrication, and in the following sections, the 10 cm × 10 cm sized device was used for the investigations.

The two different states of the bare ITO (with/without the metal deposition) and the two states of the PB film (blue/transparent) provide three different combination and four types of states. The switch of the device states is achieved by connecting the electrodes to an electrochemical work station, which works as the power source. The color states of the electrode, the transmittance spectra and the corresponding color images of the as‐assembled 10 cm × 10 cm device are all summarized in Figure 3d–f. Initially, without any connections, the device with bare ITO and the blue PB film show a blue color. By connecting the Cu‐frame to the PB film and applying an external voltage of 1.2 V, the PB film was fully oxidized and the device shows a transparent state. By connecting the bare ITO and the Cu‐frame and applying a voltage of 1.2 V, metal ions deposited on the ITO film (the PB film is blue). By observing the device from the PB film, the whole device shows a black appearance. By observing from the opposite direction, the ITO with deposited metal layers appears as a mirror with large reflectivity.

It is well known that traditional electrochromic smart window can adjust the indoor temperature by modulating the light transmittance, so as to achieve energy saving.^[^ ^22^ ^]^ Then, how about the SWH smart window with a water flow system?

The energy from solar radiation (*E*) falling onto a window will be transmitted (*E*_trans_), absorbed (*E*_abs_), and reflected (*E*_ref_).^[^ ^8^, ^14^ ^]^ Conservation of the total energy in the solar radiation beam follows Equation (1)

(1) $$E=E_{\mathrm{trans}}+E_{\mathrm{abs}}+E_{\mathrm{ref}}$$

For the electrochromic smart windows, the transmittance of the glass panes can be effectively tuned, and the transmitted energy *E*_trans_ and absorbed solar energy *E*_abs_ can be modulated on demand. For the absorbed solar energy (*E*_abs_), it mainly emitted to the surroundings (*E*_emit_, to air and water), *E*_emit_ = *E*_air_+ *E*_water_, and with the water flow system, the energy irradiated to water can be rapidly transferred, which prohibits the temperature increase and reduces the irradiation to air (*E*_air_), so that energy storage and temperature control can be simultaneously achieved by the SWH windows.

**Figure 4:**
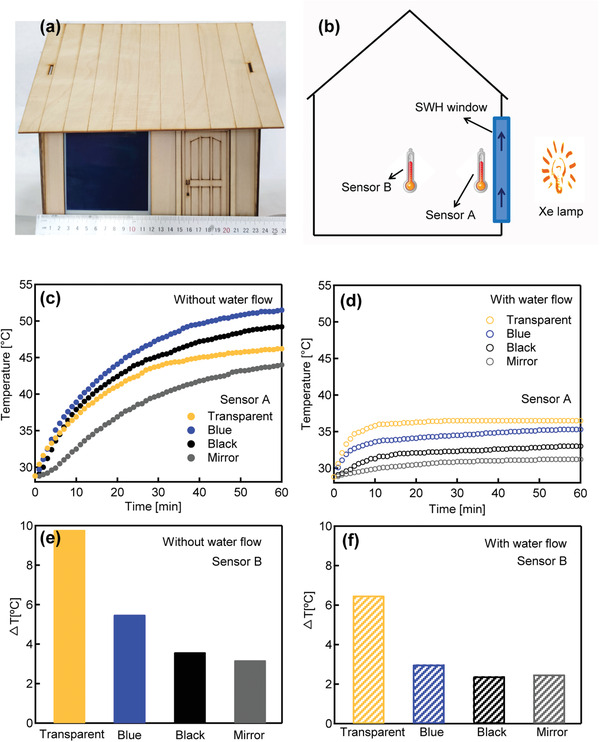
a) Photo of as‐assembled model house. b) Schematic showing the positions of the sensors in the model house. c) Temperature monitored by sensor A without water flow in 60 min. d) Temperature monitored by sensor A with water flow in 60 min. e) Temperature increase monitored by sensor B without water flow. f) Temperature increase monitored by sensor B with water flow.

To test the temperature control ability of the SWH window, as‐prepared SWH smart window was installed on a model house (22 cm × 26 cm × 26 cm, **Figure** **4**a, the geometry of the model house is provided in Figure S0, Supporting Information) and one temperature probe (sensor A) is placed close to the window to monitor the temperature increase upon the thermal radiation from the window, one temperature probe is placed in the center of the model house (sensor B) to monitor the change of the room temperature (Figure 4b). An Xe‐lamp with an air mass 1.5 global (AM 1.5 G) filter was used to simulate the solar light and placed outside the model house (≈15 cm away from the window), and the temperature increase in the house was in situ monitored by the temperature probes. For as‐assembled SWH window, the window states and the water flow provide eight types of typical combinations for modulation of the light transmittance and indoor temperature: transparent/blue/black/mirror window without water flow, and transparent/blue/black/mirror window with water flow. As shown in Figure 4c, for the window without water‐flow in the transparent/blue/black/mirror states, temperature monitored by sensor A increased rapidly from 28 to 43–53 °C within 1 h. Meanwhile, for the window with water‐flow in the transparent/blue/black/mirror states, temperature monitored by sensor A only increased from 28 to 32–36 °C within 1 h (Figure 4d), indicating ≈70% of the thermal energy emitted from the window can be moved away by the water flow system. For sensor B, when the window in the transparent/blue/black/mirror state, without water‐flow, temperature increased by 3.5–9.8 °C (Figure 4e). With the water‐flow, the temperature only increased by 2.4–6.5 °C (Figure 4f). For the windows with water‐flow, the heat on the window glass is transferred away by the flowing water, which promotes the indoor temperature modulation. One interesting observation is that the thermal radiation from the window panes is different for window in the black state and the transparent state. As shown in Figure S7 in the Supporting Information, within 80 min, the device in black state and in transparent state were all naturally cooled to room temperature, while the initial temperature for the black window is 60.1 °C and for the transparent window 50.5 °C. These results indicate the thermal irradiation from the black window is faster than the transparent window, owing to the deposited metal layer from the black window.

**Figure 5:**
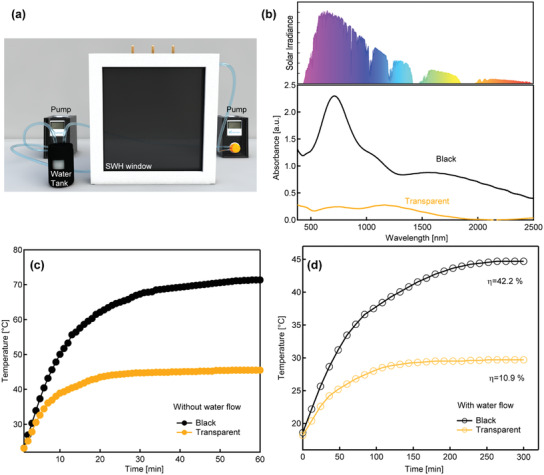
a) Schematic illustration of the solar energy harvesting by the SWH window. b) The UV–vis–NIR spectra for the SWH window in black state and transparent state. c) The temperature increase of the SWH window without water flow under simulated solar irradiation. d) The temperature increase of the electrolyte in the water tank with water flow.

Other than indoor temperature modulation, another important function of the SWH smart window is solar energy harvesting. In the following part, instead of installing the SWH smart window on a model house, we used the SWH smart window alone to investigate the function for solar energy harvesting and thermal energy storage. As shown in **Figure** **5**a and Figure S8 in the Supporting Information, the SWH window is connected to a water tank by plastic tubes, two peristaltic pumps are used for the electrolyte circulation, 100 mL electrolyte were stored in the water tank. Unlike traditional water‐flow windows using transparent glass with low absorbance, the SWH smart window show tunable absorbance upon RME. As shown in Figure 5b, with the metal deposition and the PB layer in the blue state, the SWH window is black, and the UV–vis–near IR absorption curve are significantly increased compared with the window in transparent state. The shape of the absorbance spectrum fits well with the spectrum of the solar irradiance, which indicates the effective absorption of the solar irradiation in the whole UV–vis–NIR range.^[^ ^23^ ^]^

A thermometer was placed inside the electrolyte in the window to monitor the temperature of the window. An Xe‐lamp with an AM 1.5 G filter was placed 15 cm away from the smart window as simulated solar light with an average energy density of 89.4 mW cm^−2^. As shown in Figure 5c, without the water flow, for the SWH window in both the transparent and the black state, the monitored window temperature rapidly increased in the initial 20–30 min, then the SWH window slowly reached a thermally balanced state within 1 h with no large temperature change, and in such a thermodynamical equilibrium the absorbed solar energy is equal to the energy emitted to the surroundings, *E*_abs_ = *E*_emit_, which indicates the window irradiating the collected solar energy to the surroundings and cannot be used for solar energy harvesting.^[^ ^24^ ^]^

The mean solar to heat (STH) conversion efficiency (*η*_con_) of the SWH window over the testing period is given by the ratio of the amount of thermal energy collected *Q*_col_ to the total solar energy *Q*_sol_ and is shown in Equation (2).^[^ ^15^ ^]^

(2) $$\eta_{\mathrm{con}}=\frac{Q_{\mathrm{col}}}{Q_{\mathrm{sol}}}\;=\frac{m_{w}C_{e}\left(T_{f}-T_{i}\right)}{I_{\mathrm{avg}}A_{\mathrm{sol}}\Delta t}$$

Here, *m*_w_ =* ρV*_T_ is the mass of electrolyte in the water tank (kg), *ρ* is the water density (kg m^−3^), *V*_T_ is the volume of the electrolyte in the tank (m^3^), *C*_e_ is the specific heat capacity of the electrolyte (kJ kg^–1^ K^–1^) and *T*_i_ and *T*_f_ (°C) are the initial and final temperature of the stored electrolyte, respectively. *I*_avg_ is the average light intensity (W m^−2^) as measured by the pyranometer, *A*_sol_ is the area of the SWH window (m^2^) and Δ*t* is the collection period (seconds) under the simulated solar irradiation. To determine the best flow rate, for every 12 min, 8, 15, 25, and 35 mL of the electrolyte were pumped from the tank to the SWH window, and the temperature of the water tank was in situ monitored. As shown in Figure S9 in the Supporting Information, for smart window in the black state and the transparent state, the faster the flow rate, the more rapid the window reached a balanced state. For flow rate of 8 mL and 15 mL per 12 min, the temperature increase is slow. For 25 mL and 35 mL per 12 min, the results are similar, while 35 mL per 12 min requires more energy. As a result, we used 25 mL per 12 min as the flow rate. As shown in Figure 5d, for the smart window in the transparent state, the temperature of the electrolyte in the water tank increased from 18 to 30 °C within 300 min, and the calculated STH energy conversion efficiency in 300 min is 10.9%. For the smart window in the black state, the temperature of the electrolyte increased to 45 °C, and the calculated STH energy conversion efficiency in a time period of 300 min is 42.2%, which is about four times better than the transparent window—the design for the traditional water flow windows. Here we want to emphasize that the energy conversion efficiency is not fully optimized, strategies such as adding antireflective films, reducing the thermal irradiation from the connecting tubes and adding coating to the tank may further improve the STH efficiency.

One concern of the SWH window is that the electrolyte is used instead of pure water, which may reduce the heat capacity and induce corrosion problems. Here, the main content of the electrolyte is ZnSO_4_ (1 m), the calculated heat capacity of the solution is 3.9 kJ kg^–1^ K^–1^, which is slightly reduced compared with pure water (4.2 kJ kg^–1^ K^–1^) (details for the heat capacity of the electrolyte calculation are provided in the Supporting Information).^[^ ^25^ ^]^ We did not observe noticeable corrosion of the conductive substrate or the tank during our test. As a matter of fact, the aqueous ZnSO_4_ solution is highly stable and can be used as corrosion inhibitors.^[^ ^26^, ^27^ ^]^ Another concern is that owing to the self‐discharging process, it is essential to apply a bias to maintain the deposited metal layer. First, the power to maintain the metal deposition is ultralow (≈100–200 nW cm^−2^ for 12 h), which is negligible. Second, the SWH window can be connected with a piece of solar cell (Figure S10, Supporting Information), which provide the essential bias of ≈1 V to maintain the black state.

**Figure 6:**
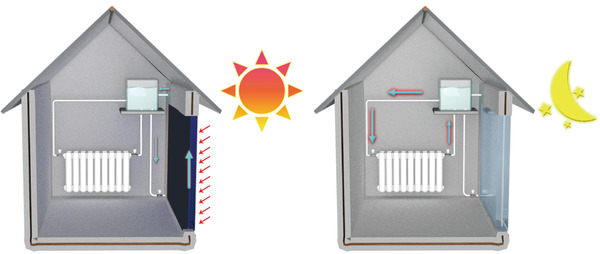
Schematics showing the energy harvesting/temperature tuning of the SWH window between day and night.

The above discussions indicate the SWH smart windows can effectively harvest the solar energy, and the stored thermal energy may potentially be used for low‐temperature floor/wall heating. One interesting question is that, does this kind of design have any chance to be used in a real world? Nowadays, there are lots of areas with large temperature differences between day and night. For example, in the Sahara, in the Middle East, and some areas in the northwest part of China, the temperature at noon can be as high as 50 °C and at night the temperature can drop to below 10 °C.^[^ ^28^ ^]^ In such areas, the indoor cooling in the daytime and the warming at night are both important. As‐designed SWH smart windows can effectively reduce the indoor solar irradiation in the day time and store the thermal energy by heating water for indoor warming at night (**Figure** **6**), which may potentially be used in these areas, and achieve indoor comfort with much lower energy cost. Currently, our study only proposed the basic design of the SWH smart windows with highly simplified analytical models, to achieve the goal of practical applications, comprehensive models of solar water heating smart windows, their integration into buildings and their large‐size applications in real world require further investigations.^[^ ^29^, ^30^ ^]^

## 3 Conclusions

Here, a new design of solar water heating smart window with the integration of the traditional electrochromic window and a water‐flow system is proposed. The reversible metal electrodeposition on transparent conductive substrates not only provides modulation of the window transmittance but also works as a flat‐plate heat collector for solar energy collection. Light transmittance, room temperature control and energy collection/storage can all be simultaneously modulated in the SWH window, which provides energetic and functional advantages over the traditional electrochromic windows and the water‐flow windows. Our research may bring new insights in the design of next‐generation green buildings.

## Conflict of Interest

The authors declare no conflict of interest.

## Supporting information

Supporting Information

## Data Availability

The data that support the findings of this study are available in the supplementary material of this article.
