# Supplementary material for: A Solar Water‐Heating Smart Window by Integration of the Water Flow System and the Electrochromic Window Based on Reversible Metal Electrodeposition
Source: Adv Sci (Weinh). 2021 Dec 28;9(6):2104121. doi: 10.1002/advs.202104121 (PMC8867160; doi:10.1002/advs.202104121)
Supplement: Supplementary file 1 — Supporting Information [file ADVS-9-2104121-s001.pdf]

ADVANCED SCIENCE
Open Access

Supporting Information

for *Adv. Sci.,* DOI: 10.1002/advs.202104121

A Solar Water-heating Smart Window by Integration of the Water Flow System and the Electrochromic Window Based on Reversible Metal Electrodeposition

Ling Wang, Xiuling Jiao, Dairong Chen and Ting Wang**

Supporting Information

A solar water-heating smart window by integration of the water flow system and the electrochromic window based on reversible metal electrodeposition

Ling Wang, Xiuling Jiao, Dairong Chen and Ting Wang**

School of Chemistry& Chemical Engineering, National Engineering Research Center for Colloidal Materials, Shandong University
E-mail:t54wang@sdu.edu.cn; cdr@sdu.edu.cn

1. Materials

Zinc sulfate heptahydrate ($ZnSO_4 \cdot 7H_2O$, ≥99.95 %), copper (II) chloride ($CuCl_2$, ≥99.99 %) and potassium chloride (KCl, 99.8%) were purchased from Sigma-Aldrich. Iron chloride hexahydrate ($FeCl_3 \cdot 6H_2O$, 99 %), potassium ferricyanide ($K_3[Fe(CN)_6]$, ≥99.0%) and copper foil (Cu, 99.9 %) were purchased from Aladdin Shanghai Aladdin Biochemical Technology Co. Ltd.. Tin-doped indium oxide (ITO) glasses (10*10*0.12 mm, unpolished float glass, SiO_2 passivated, 7Ω) were purchased from Foshan yuanjingmei Glass Co. Ltd.. All the reagents are of analytical grade and used as-received without further purification.

2. Characterization

The crystal structures of the metal and PB films were examined by X-ray diffraction (XRD, Rigaku D/Max 2200PC diffractometer with a graphite monochromator and Cu Kα radiation (λ= 0.15418 nm)). The morphology and microstructure of the metal and PB films were characterized by the field emission scanning electron microscope (FE-SEM, SU8010) equipped with an energy dispersive X-ray spectroscopy (EDS, XFlash 6160). Electrochemical measurements were obtained using an electrochemical workstation (CHI 660E).

3. Prussian blue film preparation

ITO glass substrates were pre-treated by acetone, ethanol, deionized water sequentially and dried in ambient air. The precursor solution was obtained by mixing

50 mmol/L KCl, 10 mmol/L $FeCl_3$ and 10 mmol/L $K_3[Fe(CN)_6]$ in deionized water under stirring for 5 min at room temperature. Then the electrodeposition procedure was carried out in a three-electrode cell system with the ITO glass as the working electrode, Ag/AgCl electrode as the reference electrode and Pt as the counter electrode. Typically, a constant current density of 20 μA cm^{-2} was applied to the electrodeposited system and the optimal deposition time was 600 s. After the deposition process, the PB electrodes were washed by deionized water and dried at 60 °C for 1 h in the oven.

4. Calculation of the ion diffusion coefficients for the PB film and explanation for the performance fluctuation of the PB film

To further investigate the performance fluctuation of the PB film, we have calculated the diffusion coefficients D (cm^2/s) of Zn^{2+} and Cu^+ in the PB using CV results in Figure S4 by the Randles-Sevcik equation:

$$I_p = kn^{3/2}AD^{1/2}cv^{1/2} \text{ (Equation S1)}$$

where I_p (A) is the peak current, k is the Randles-Sevcik constant, $k = 2.69\times10^5$, n is the transferred electron number involved in the redox process, A (cm^2) is the area of the electrode, C (mol/cm^3) is the concentration of ion in the bulk solution, and v (V s^{-1}) is the scan rate.

Ion diffusion coefficients:

$$D_{(initial)} = \left(\frac{I_p}{v^{\frac{1}{2}}} \times \frac{1}{k \times n^{\frac{3}{2}} \times A \times C}\right)^2 = \left(0.275 \times \frac{1}{2.69\times10^5 \times 2^{\frac{3}{2}} \times 3 \times 1}\right)^2 = 1.44 \times 10^{-14} \text{ cm}^2\text{/s}$$

$$D_{(200)} = \left(\frac{I_p}{v^{\frac{1}{2}}} \times \frac{1}{k \times n^{\frac{3}{2}} \times A \times C}\right)^2 = \left(0.083 \times \frac{1}{2.69\times10^5 \times 2^{\frac{3}{2}} \times 3 \times 1}\right)^2 = 1.6 \times 10^{-15} \text{ cm}^2\text{/s}$$

$$D_{(1000)} = \left(\frac{I_p}{v^{\frac{1}{2}}} \times \frac{1}{k \times n^{\frac{3}{2}} \times A \times C}\right)^2 = \left(0.082 \times \frac{1}{2.69\times10^5 \times 2^{\frac{3}{2}} \times 3 \times 1}\right)^2 = 1.56 \times 10^{-15} \text{ cm}^2\text{/s}$$

As shown in the above calculations, the initial ion diffusion coefficient D dropped from 1.44×10^{-14} cm^2/s to 1.6×10^{-15} cm^2/s after 200 cycles, which is about 10 times dropped and induces the rapid performance decay within 200 cycles. After 1000 cycles, the diffusion coefficient only slightly decays from 1.6×10^{-15} cm^2/s to 1.56×10^{-15} cm^2/s. Such a small change of the ion diffusion coefficient cannot greatly influence the performance of the film. Then, why the PB performance

increased from 200 cycles to 1000 cycles? We further obtained the SEM images of the PB film in the initial state before cycling, after 200 cycles and 1000 cycles. As shown in Figure S5, with continuous cycling, the PB film experiences gradual etching, and the relatively clean film etched to rough surface within 200 cycles, and after 1000 cycles, the film becomes highly porous. We propose the increased performance of the film may be owing to the highly porous surface of the film, which increase the active surface area and promotes the color switching process.

5. Solar water-heating smart window assembly

Solar water-heating smart window was assembled using two pieces of ITO glass, one was bare ITO glass and another was with PB deposited on it. We prepared two sizes of devices, one size is 5*5*0.5cm^3, which used for the electrochromic measurements and solar energy harvesting system tests. Another is 10*10*0.5 cm^3, used for the test of indoor temperature control. The Cu metal frame was used as the anode, and aqueous electrolyte containing $ZnSO_4$(1 M)/$CuCl_2$(0.01 M) were used as the electrolyte. As shown in Figure S2, first, the two ITO electrodes and the Cu metal frame (in the middle of the two ITO electrodes) were inserted into a custom resin (photosensitive resin 9400) box, assembled as a cell. Epoxy glue was used to seal three sides of the cell. The electrolyte was injected into the cell by a syringe through the unsealed side of the cell. Before injection, the empty cell and the electrolyte in a vial were purged with dry N_2 for 15 min to drive away ambient air. Finally, the cover matching with the resin frame was encapsulated on the cell, and the solar water-heating smart window was finally prepared.

6. Temperature control and solar energy harvesting test

Temperature control: The temperature control test was carried out in a 22*26*26 cm^3 model house, which was made of wood. In order to avoid the influence of ambient temperature as much as possible, we attached a layer of insulation cotton to the interior of the house. The photo of the model house is shown in Figure 4a. The size of the window is 10*10 cm^2, which corresponds to the size of the device. In the temperature control test, we tested the air temperature near the window (position A) and the air temperature in the middle of the model house (position B), respectively. The model of the thermometer is GJD-200LED, which was purchased from Hengshui Zhengxu Electronic Technology Co. Ltd.. The Xenon lamp with filter was used as the light source to simulate the sunlight and it was placed 15 cm away from the window

outside the model house and adjusted the optical power density to that the average optical power density of the device was 84 mW/cm^2 (the center of the window was 100 mW/cm^2 and the edge of the window was 68 mW/cm^2). The temperature of position A and B were measured continuously and the temperature changes were recorded every 1 minute. To maintain the metal deposition of the SWH window, a bias of 1 V between the ITO and Cu-frame was applied to the SWH window in the temperature control measurements.

The details of the model house: The geometry of the model house is provided in Figure S0 in the SI. The material used in the model house is wood with a thickness of 0.3 cm and a thermal conductivity (λ) of 0.1 W/(m · K). According to the relationship between thermal conductivity and heat transfer coefficient (K): d/λ=R (d is the thickness of the material, R is thermal resistance),1/R=K, K= λ/d. The calculated heat transfer coefficient is 33.3 W/(m^2 · K). The emissivity of the walls is 0.96. In order to improve the thermal insulation, we have covered a polyurethane (PU) foam inside the wall of the house to improve the thermal insulation. The thickness of PU foam is 0.5 cm, the thermal conductivity is 0.022~0.033 W/(m · K).

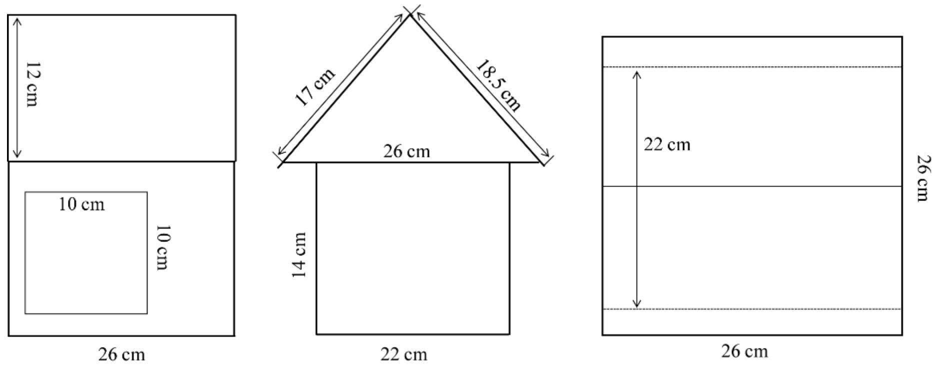

Figure S0. The geometry of the model house.

Solar energy harvesting: The solar energy harvesting test was shown in Figure 5a, the solar energy harvesting system was assembled using two peristaltic pumps of model STP-F01A, a vacuum cup as water tank (with 100 mL electrolyte) and a thermometer placed inside (GJD-200LED). An Xenon lamp with filter was used to simulate the solar light and placed 15 cm away from the device. Peristaltic pumps provide the power for the electrolyte circulation. The average optical power density of the device was adjusted to 89.4 mW/cm^2 (the center position of the window is 100 mW/cm^2, and the edge position is 78.8 mW/cm^2), and the device was continuously

tested and the temperature changes in the vacuum cup were recorded for 4 hours. In the test, the flow rate of the peristaltic pump was set at 25 mL/min and the cycle was performed every 12 minutes. The thermometer was placed in the vacuum cup to test the temperature change and the temperature was recorded every 12 minutes. To maintain the metal deposition of the SWH window, a bias of 1 V between the ITO and Cu-frame was applied to the SWH window in the solar energy harvesting measurements.

The details of the STP-F01A peristaltic pump: the motor voltage is 24 V, the rated voltage is 12 W, and the output DC is 24 V, 1.9 A. According to the calculation of power consumption formula W= P×t, the power consumption of two peristaltic pumps is W= 2 ×12 W×(5/60) h = 2 Wh. The heat generated in the circulation process is very small, which has no effect on the temperature recorded in the experiment and can be ignored.

7. Specific heat capacity of the electrolyte

For the calculation of specific heat capacity of multicomponent system, the following formula shall be followed:

$$C_P = C_L X_L + C_S X_S \qquad \text{(Equation S1)}$$

Here, C_P is the specific heat capacity of solvent (J kg^{-1} K^{-1}), X_L is the mass fraction of solvent, C_S is the specific heat capacity of solute (J kg^{-1} K^{-1}), X_S is the mass fraction of solute.

The specific heat capacity of the electrolyte:

$$C_P = C_L X_L + C_S X_S$$

$$= C_L \times \frac{m_{solvent}}{m_{solvent}+m_{solute}} + C_S \times \frac{m_{solute}}{m_{solvent}+m_{solute}}$$

$$= 4.2 \times 10^3 \times \frac{100}{100+28.7} + 2.8 \times 10^3 \times \frac{28.7}{100+28.7}$$

$$= 3.9 \times 10^3 \text{J} \cdot \text{Kg}^{-1} \cdot \text{K}^{-1}$$

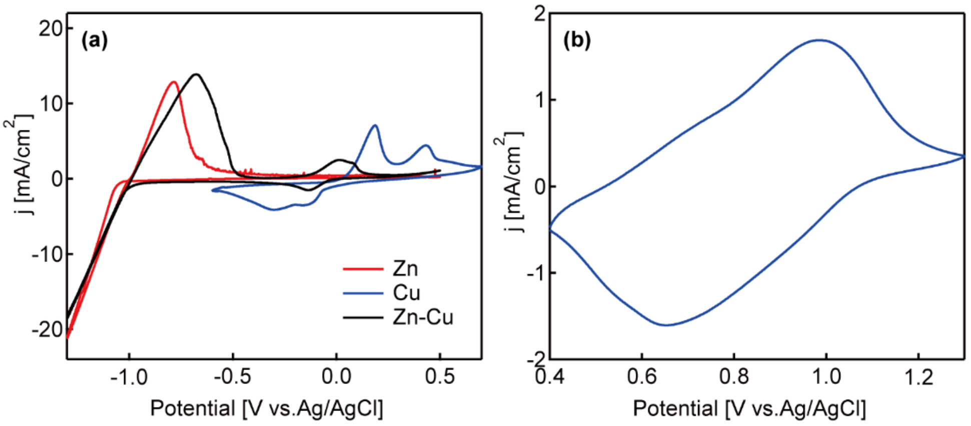

Figure S1. (a) Cyclic voltammograms of Zn (red), Cu (blue), and Zn-Cu (black) on ITO glass in a three-electrode cell. (b) Cyclic voltammograms of the PB film (blue) in a three-electrode cell.

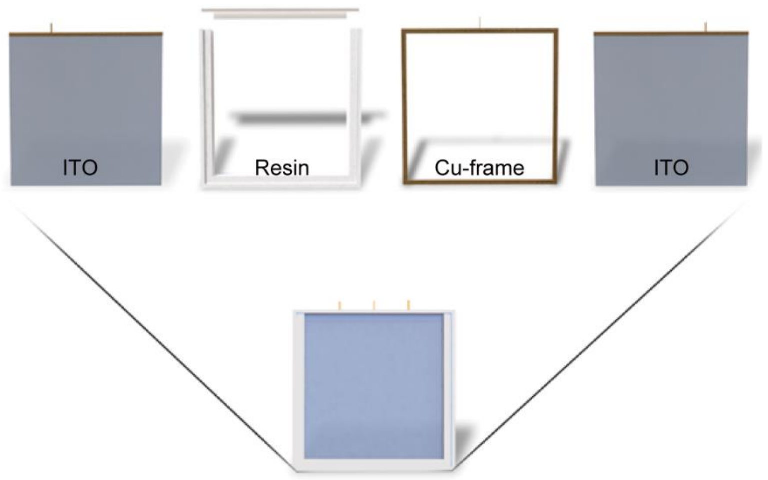

Figure S2. Schematic illustration of the window architectures.

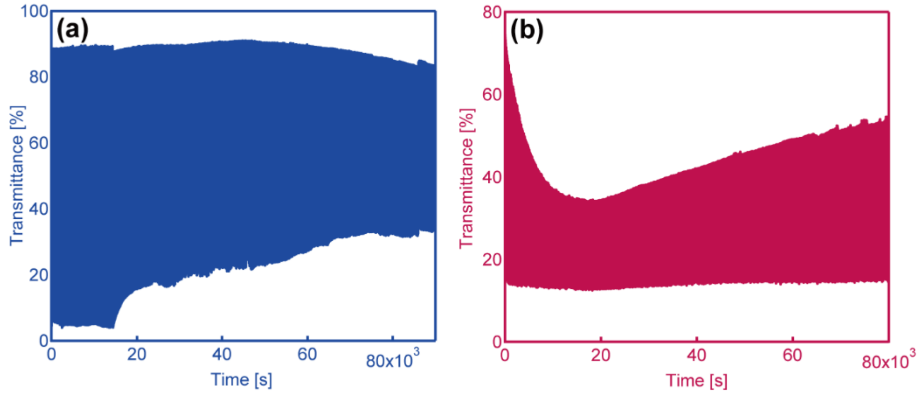

Figure S3. Optical transmittance of as-assembled SWH window over a course of 1000 cycles for (a) bare ITO with RME, (b) PB film.

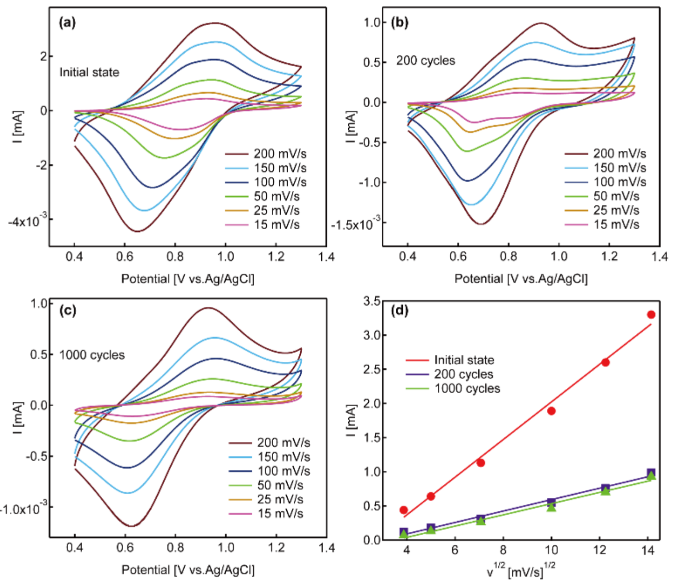

Figure S4. Cyclic voltammograms of the PB film (a) in the initial state, (b) after 200 cycles and (c) after 1000 cycles at various scan rates. (d) Anodic peak current of the PB film obtained from the cyclic voltammograms in the initial state, after 200 cycles and 1000 cycles.

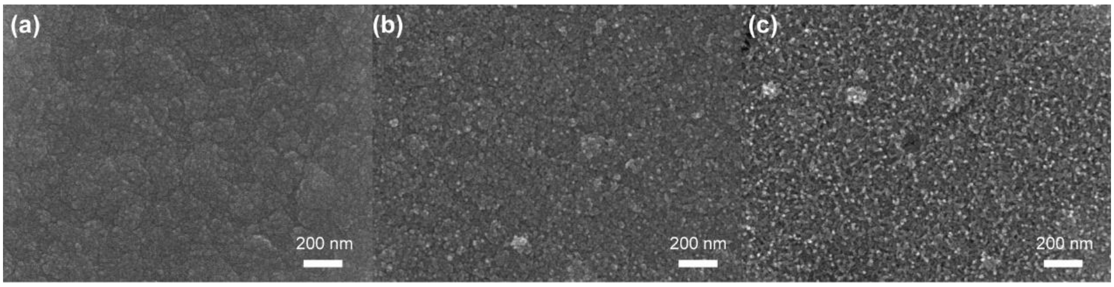

Figure S5. SEM images showing the surface morphology of the PB film (a) in the initial state, (b) after 200 cycles and (c) after 1000 cycles.

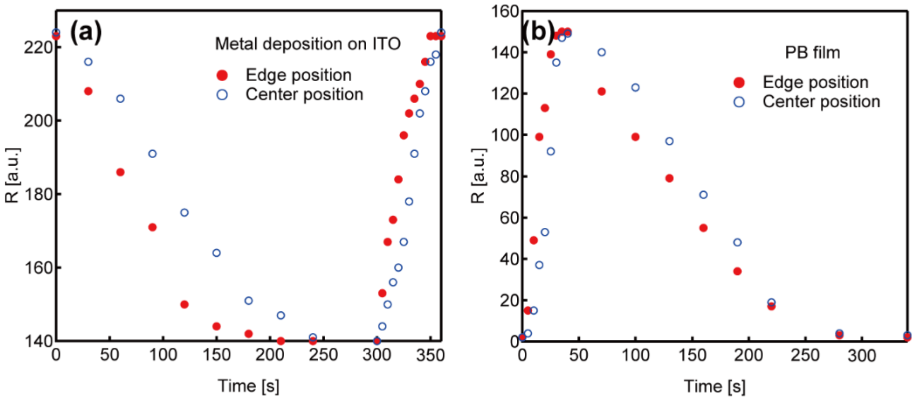

Figure S6. (a) R-value (red) change curves for the RME process with transmittance switching at voltages of 1.0 V (stripping, from 0 s to 250 s) and -1.2 V (deposition, from 300 s to 350 s). (b) R value change curves for the PB film with transmittance switching at voltages of 1.3 V (blue-transparent, 0 s to 50 s) and 0.4 V (transparent-blue, 50 s to 350 s).

Since the 10 cm×10 cm device is too large to fit into the UV-vis spectrometer for continuous measurement, the switching time of the 10 cm×10 cm device was tested by continuously monitoring the RGB (red, green, blue) changes of the images. It is well known that due to the potential drop of ITO itself, the color change between the middle and the edge will be out of sync and there will be a delay in the middle in the color change process of large-area devices. Therefore, we recorded the continuous RGB change between the edge and the central part in the color change process (Table S1 and Table S2), and selected the R value for drawing, so as to calculate the color response time of large-area devices, as shown in Figure S6.

Table S1. Recorded value of RGB in continuous time of RME process

	Edge position			Center position		
Time /s	R	G	B	R	G	B
0	223	226	216	224	225	218
30	208	210	205	216	219	210
60	186	191	185	206	206	202
90	171	183	177	191	190	185
120	150	148	139	175	175	168
150	144	139	130	164	165	160
180	142	142	138	151	153	150
210	140	139	142	147	149	151
240	140	139	142	141	139	142
300	140	139	142	140	139	142
305	153	160	160	144	146	148
310	167	173	170	150	151	148
315	173	174	170	156	156	154
320	184	190	184	160	158	154
325	196	199	194	167	159	155
330	202	203	194	178	176	172
335	206	204	198	191	195	188
340	210	206	198	202	202	196
345	216	219	210	208	204	198
350	223	226	216	216	217	210
355	223	226	216	218	219	211
360	223	226	216	224	225	218

Table S2. Recorded value of RGB in continuous time for PB film color change process

	Edge position			Center position		
**Time /s**	**R**	**G**	**B**	**R**	**G**	**B**
0	2	90	191	2	90	191
5	15	98	178	4	94	189
10	49	109	169	15	103	182
15	99	133	160	37	116	171
20	113	133	151	53	119	161
25	139	148	140	92	129	153
30	148	159	138	135	147	146
35	150	160	138	147	159	139
40	150	160	138	149	160	138
70	121	141	146	140	156	140
100	99	134	152	123	142	146
130	79	129	162	97	130	154
160	55	120	169	71	121	161
190	34	116	173	48	112	172
220	17	102	184	19	105	186
280	3	91	191	4	90	190
340	2	91	191	3	91	191

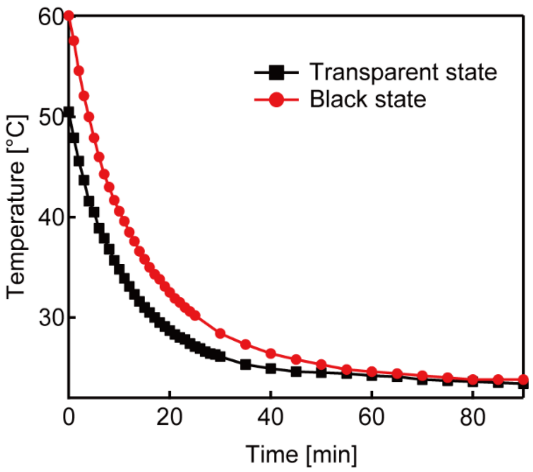

Figure S7. Comparison of the thermal radiation from the window panes for the smart window in the black state and the transparent state.

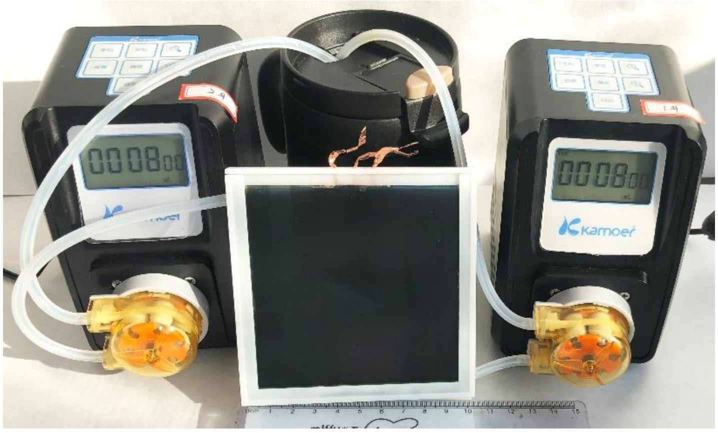

Figure S8. Photo showing the assembled electrochromic window with the water flow system.

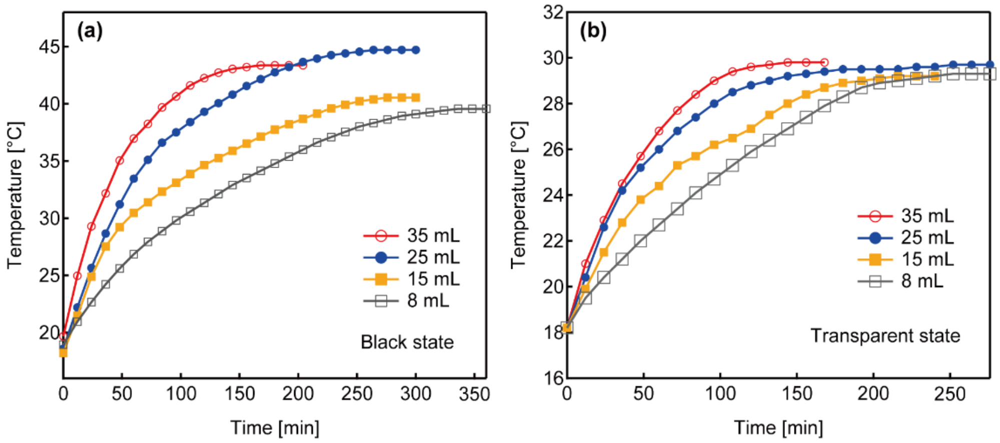

Figure S9. (a) The temperature increase with different water flow rate for the smart window in the black state. (b) The temperature increase with different water flow rate for the smart window in the transparent state. The flow rates were set at 8 mL, 15 mL, 25 mL and 35 mL per 12 min.

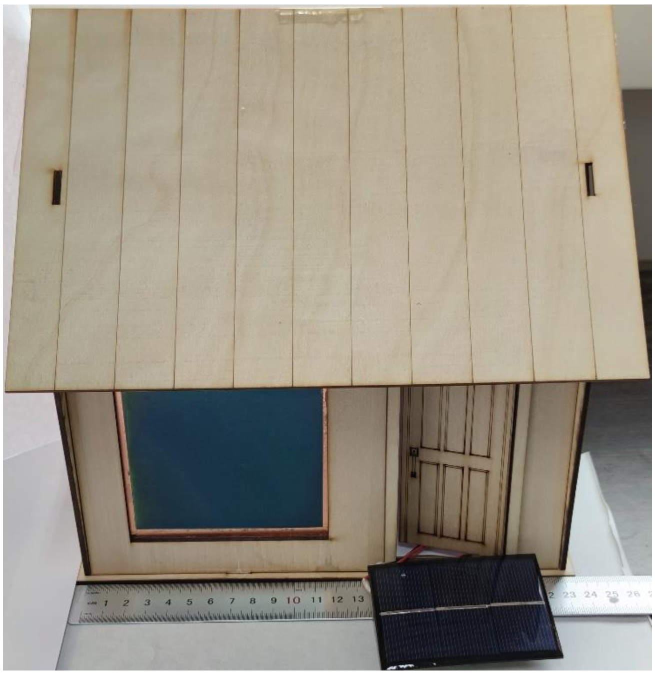

Figure S10. SWH window on a model house with connected PV module. The PV with an open voltage of 1.1 V, which can provide the power essential for maintain the deposited metal layers.
